# Effects of a High-Fat Diet on Tissue Mass, Bone, and Glucose Tolerance after Chronic Complete Spinal Cord Transection in Male Mice

**DOI:** 10.1089/neur.2020.0014

**Published:** 2020-07-23

**Authors:** Zachary A. Graham, Xin-hua Liu, Lauren Harlow, Jiangping Pan, Daniella Azulai, Hesham A. Tawfeek, Russell D. Wnek, Alex J. Mattingly, William A. Bauman, Joshua F. Yarrow, Christopher P. Cardozo

**Affiliations:** ^1^ Research Service, Birmingham VA Medical Center, Birmingham, Alabama, USA.; ^2^ Department of Cell, Developmental and Integrative Biology, University of Alabama-Birmingham, Birmingham, Alabama, USA.; ^3^ Center for the Medical Consequences of SCI, James J. Peters VA Medical Center, Bronx, NY, USA.; ^4^ Icahn School of Medicine at Mount Sinai, New York, NY, USA.; ^5^ Research Service and Brain Rehabilitation Research Center, Malcolm Randall VA Medical Center, North Florida/South Georgia Veterans Health System, Gainesville, Florida, USA.; ^6^ Division of Endocrinology, Diabetes, and Metabolism, University of Florida College of Medicine, Gainesville, Florida, USA.

**Keywords:** high-fat diet, metabolism, paralysis, spinal cord injury, type 2 diabetes

## Abstract

Spinal cord injury (SCI) is associated with obesity and is a risk factor for type 2 diabetes mellitus (T2DM). Immobilization, muscle atrophy, obesity, and loss of sympathetic innervation to the liver are believed to contribute to risks of these abnormalities. Systematic study of the mechanisms underlying SCI-induced metabolic disorders has been limited by a lack of animal models of insulin resistance following SCI. Therefore, the effects of a high-fat diet (HFD), which causes weight gain and glucose intolerance in neurologically intact mice, was tested in mice that had undergone a spinal cord transection at thoracic vertebra 10 (T10) or a sham-transection. At 84 days after surgery, Sham-HFD and SCI-HFD mice showed impaired intraperitoneal glucose tolerance when compared with Sham control (Sham-Con) or SCI control (SCI-Con) mice fed a standard control chow. Glucose tolerance in SCI-Con mice was comparable to that of Sham-Con mice. The mass of paralyzed skeletal muscle, liver, and epididymal, inguinal, and omental fat deposits were lower in SCI versus Sham groups, with lower liver mass present in SCI-HFD versus SCI-Con animals. SCI also produced sublesional bone loss, with no differences between SCI-Con and SCI-HFD groups. The results suggest that administration of a HFD to mice after SCI may provide a model to better understand mechanisms leading to insulin resistance post-SCI, as well as an approach to study pathogenesis of glucose intolerance that is independent of obesity.

## Introduction

Several cross-sectional studies over the past 25 years have indicated that persons with spinal cord injury (SCI) are more likely to develop glucose intolerance and type 2 diabetes mellitus (T2DM) than age-matched able-bodied controls and that motor-complete SCI and tetraplegia confer greater relative risk as compared with motor incomplete injuries or paraplegia.^1–3^ Larger, survey-based studies of the SCI population have provided independent confirmation of these observations.^4,5^ Several physiological mechanisms have been proposed for the predisposition to disorders of fat and carbohydrate metabolism after SCI based upon the current understanding of the pathogenesis of T2DM in the general population.

Determinants for insulin resistance and carbohydrate disorders in the general population include obesity, more specifically visceral adiposity, and inactivity,^6–11^ both of which are more prevalent in the SCI population^12^; an additional determinant in the SCI population includes a marked reduction in muscle mass, which is pertinent given that over 60% of insulin-stimulated glucose uptake occurs in skeletal muscle.^7,9,10^ Another risk factor is sublesional osteoporosis, which may be relevant to glucose homeostasis, because bone is a site of glucose disposal due to ongoing bone remodeling throughout the lifespan^13^ (albeit less so with advancing age) and because bone produces osteocalcin, a molecule that stimulates insulin secretion from pancreatic β cells as well as glucose and fatty acid metabolism in muscle.^14^ Lastly, for those with higher spinal injury levels, it has been suggested that, in rats as well as in humans, impaired sympathetic innervation of the liver and abdominal fat compartment may contribute to changes in fat metabolism and/or glucose intolerance.^15,16^ Very little else is known about precise physiological, cellular, or molecular mechanisms responsible for the predisposition of those with SCI to insulin resistance and T2DM.

Animal models of T2DM resulting from leptin deficiency, leptin insensitivity, or diets high in long chain fatty acids have been widely used to understand the contribution of humoral, cellular, and molecular factors to the development of T2DM.^17^ We reasoned that a diet high in long chain fatty acids, a well-known model of diet-induced glucose intolerance,^17^ might amplify or induce glucose intolerance in rodents after SCI, and thus provide a model that could be used to further delineate the various cellular and molecular factors that contribute to the heightened risk of developing T2DM in those with SCI. The present study aimed to understand how feeding male mice a diet containing 60% lard after a complete T10 spinal cord transection would influence glucose tolerance, the mass of muscle, liver, and adipose tissue deposits, and parameters of bone microarchitecture.

## Methods

### Animals

Three-month-old male C57BL/6 mice were purchased from Charles River Laboratories. Males were selected because they comprise 80% of the general SCI population in the United States^18^ and 95% of the U.S. veteran population.^19^ In addition, prior work has demonstrated that male mice are more responsive to the deleterious effects of a high-fat diet (HFD) on body mass and glucose tolerance.^20^ The procedures for a complete transection SCI in mice have been previously described in detail.^21^ Briefly, all mice were randomly assigned to groups that underwent either T9-10 laminectomy (Sham) or laminectomy followed by complete transection SCI at T10 (SCI). We chose a T10 transection because in clinical studies persons with SCI who have a motor-complete injury have a higher likelihood of developing glucose intolerance or T2DM,^2,12^ because a transection causes reproducible and consistent motor-complete SCI, and because our group has shown low thoracic injuries promote substantial muscle, bone, and fat loss.^22^ Additionally, transection at T10 likely affects liver metabolic function as thoracic contusion SCI leads to rapid liver dysfunction.^23^ Animals were then randomly placed in a control (Con) or HFD group.

Group sizes at the time of surgery were: Sham-Con, *n* = 9; Sham-HFD, *n* = 9; SCI-Con, *n* = 8; and SCI-HFD, *n* = 10. Group sizes at the time animals were euthanized were: Sham-Con, *n* = 9; Sham-HFD, *n* = 9; SCI-Con, *n* = 5; and SCI-HFD, *n* = 7. There were six cases of premature death in the SCI groups. Bladder rupture during manual expression occurred in two animals, seizure-like movements during bladder expression in two animals, and unknown reasons in the remainder.

All surgeries were performed under deep anesthesia induced and maintained by inhalation of 2–3.5% isoflurane. Hair was removed from the skin along the spine with a clipper and the skin was cleaned with 70% ethanol and a beta-iodine solution. An incision was made from T7 to T11 and the muscle over the spinal column was peeled away by blunt dissection. The vertebral arches of T9-10 were carefully removed with sharp scissors and animals selected for the Sham group had the incision site immediately closed in layers using sutures and were then placed in a clean cage. Those animals selected for SCI had the spinal cord completely cut using surgical scissors. A micro-scalpel was passed through the transection site several times to ensure there were no intact neural tracts spanning the incision site, after which an inert gel foam was placed between the ends of the severed spinal cord. The incision site was closed in layers with sutures and the animals were placed in clean cages.

All animals were single-housed post-operatively. For the first 24 h after SCI, cages were placed on a warming pad through which water warmed to 37°C was circulated. Animals were treated with a daily injection of 5 mg/kg carprofen and 5 mg/kg/day Baytril for 3 days post-surgery. Animals were also administered 1 mL of warmed lactated Ringer's solution subcutaneously for 7 days to prevent dehydration. Bladders were manually expressed 2 to 3 times per day for the length of the study. Animals were observed at least daily for any signs of incomplete transection such as hind limb movement, spasms of hind limbs, excessive grooming of areas below the lesion, and other manifestations of pain such as autophagy. None of the animals in this study demonstrated traits of incomplete spinal cord transection. All procedures were approved by the Institutional Animal Care and Use Committee (IACUC) at the James J. Peters VA Medical Center (Protocol #: CAR-16-54).

### High-fat diet

A lard-based HFD was purchased from Research Diets (D12492; 60% fat, 20% protein, 20% carbohydrate, 5.21 kcal/g energy density). A source- and micronutrient-controlled chow with protein content identical to the HFD and a macronutrient content similar to standard rodent chow (D12450J; 10% fat, 20% protein, 70% carbohydrate, 3.82 kcal/g energy density) was fed to the diet-control group. Animals were on standard rodent chow pre-surgery with *ad libitum* access to HFD and control chows beginning immediately post-surgery. HFD chow was replaced every 3 to 4 days, whereas control chow was replaced weekly.

### Intraperitoneal glucose tolerance tests

At 56 and 84 days post-surgery, after a 4-h fast, animals received an intraperitoneal glucose tolerance test (IPGTT) by intraperitoneal injection of 2 g/kg of a 20% glucose solution. Fifty-six and 84 days were chosen as these time-points are within the expected time frame within which 3-month-old male mice develop diet-induced glucose intolerance, resulting in opportunities to study any diet-induced disruption in glucose handling in mice after SCI.^20^ Blood samples were taken from a small tail-snip and a drop of blood was placed on a glucose test strip and measured using a glucometer (AimStrip Plus, Germaine Labs). Blood glucose levels were determined before injection of the glucose solution and at 15-, 30-, 60-, and 120-min post-injection. Animals were kept in their home cages during the test period. Of note, one SCI-Con and one SCI-HFD mouse did not have values recorded due to technical issues at 56 days and were omitted from analysis, whereas two SCI-Con mice died from post-operative complications between the 56-day and 84-day tests. Area under the receiver operating characteristic curve (AUC) was calculated from the IPGTT tests using 0 as the Y-axis baseline, with X-axis values being determined by row value.

### Body and tissue mass

Body mass was determined on the day of surgery and then weekly thereafter for 12 weeks. Tissues were carefully excised from animals under deep isoflurane anesthesia then weighed before being frozen in liquid nitrogen-cooled isopentane for later analysis. Tissues collected included the soleus, plantaris, gastrocnemius, and tibialis anterior (TA) muscles; liver; and epididymal (eFat), inguinal (iFat), and omentum (oFat) adipose deposits. The femurs were cleaned of muscle and connective tissue, weighed, and stored in 70% ethanol for later analysis. For each mouse, tissue masses were normalized to pre-surgery body mass.

### Bone DEXA and microCT

The entire right femur was imaged on a MedikorsInAlyzer dual-energy x-ray absorptiometry (DEXA) system (Seongnam, South Korea; Supplementary Figure S1A) using the “Accuracy” mode to determine whole femur areal bone mineral density (aBMD; g/cm^2^), bone mineral content (BMC; g), bone area (cm^2^), and estimated bone volume (cm^3^). The same bone was also imaged on a Bruker Skyscan 1172 high-resolution microcomputed tomography (microCT) system (Kontich, Belgium) to determine cancellous and cortical bone morphology at select regions of interest (ROIs) using our previously published methods^24–27^ and as shown in Supplementary Figure S1B using the following settings: 50 kV/200 uA, 0.5-mm Al filter, 2 K resolution, 7.0 μm voxel, 0.5-degree rotation step, and 180-degree tomographic rotation. The femur was selected because it exhibits marked bone loss after SCI and because more than 80% of fractures requiring hospitalization in the SCI population occur at the distal femur.^28^ Further, we have demonstrated cancellous bone deficits are present at the distal femur in non-SCI rodents in response to high-fat and high-fat/high-sugar feeding.^24^

The microCT-based cancellous ROI began 0.5 mm proximal to the distal femoral metaphysis growth plate and encompassed 1.5 mm and the cortical ROI encompassed 1.0 mm surrounding the femoral midshaft. Outcomes of interest were distal femur: cancellous bone volume (BV/TV, %), trabecular number (Tb.N, #/mm), trabecular thickness (Tb.Th, mm), trabecular separation (Tb.Sp, mm); and femoral diaphysis: total area inside the periosteal envelope (T.Ar, mm^2^), cortical bone area (Ct.Ar, mm^2^), cortical area fraction (Ct.Ar/Tt.Ar, %), and cortical thickness (Ct.Th, mm). Additionally, cancellous volumetric bone mineral density (vBMD; g/cm^3^) and cortical volumetric tissue mineral density (vTMD; g/cm^3^) were determined at the previously defined ROIs, after calibration with hydroxyapatite phantoms.

### Statistical analysis

All statistical analyses were completed using a 2 × 2 (Surgery × Diet) two-way mixed model analysis of variance (ANOVA) with Tukey's multi-comparison post hoc tests. All data are reported as the mean ± 95% confidence interval, with the exception of body mass and IPGTT over time, which are represented as ± SEM (standard error of the mean) for clarity in the figures. Column means are shown at the bottom or top of each bar chart and meaningful differences between groups are shown with brackets and the adjusted *p*-value from the Tukey's post hoc tests. A complete listing of the results of all statistical tests and outcomes can be found in Supplementary Table S1. The threshold for significance was *p* < 0.05.

## Results

### Body mass

**FIG. 1.**
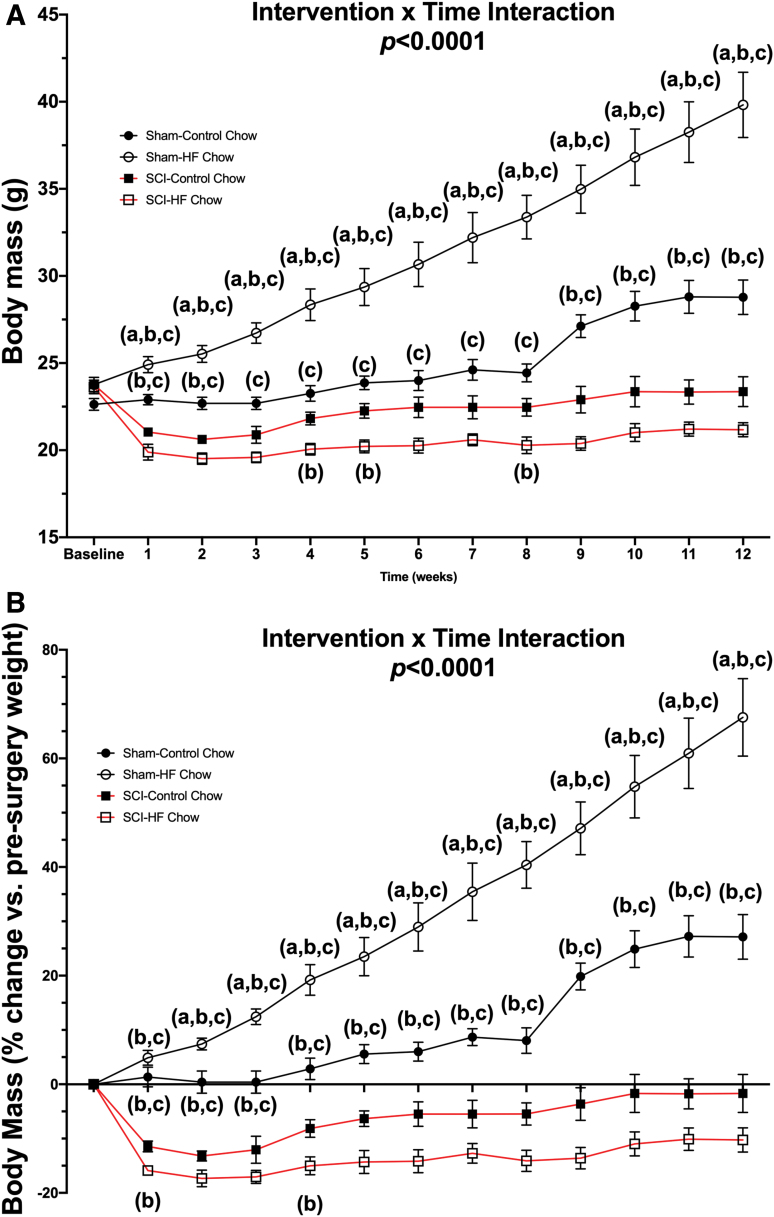
Body mass was recorded weekly and expressed in **(A)** absolute body mass and **(B)** relative mass (percent of body weight taken just prior to surgery). Significant Group × Time interaction effects were noted between groups for both absolute and relative body mass (*p <* 0.0001). Group differences as determined by Tukey's multiple comparisons post hoc at each time-point (*p* < 0.05) are denoted as (a) differences compared with Sham-Con, (b) differences compared with SCI-Con, and (c) differences compared with SCI-HFD. Data are presented as mean values ± SEM for clarity. Group sizes were: Sham-Con, *n* = 9; Sham-HFD, *n* = 9; SCI-Con, *n* = 5; and SCI-HFD, *n* = 7. All simple effects differences can be found in Supplementary Table S1. Con, control; HFD, high-fat diet; SCI, spinal cord injury; SEM, standard error of the mean.

There was a Surgery × Diet interaction effect for both absolute body mass (Fig. 1A; *p <* 0.0001) and relative percent loss in body mass (Fig. 1B; *p* < 0.0001). Post hoc testing showed a robust increase in body mass for the Sham-HFD group compared with the Sham-Con group at all time-points. There were also reductions in body mass for both SCI-Con and SCI-HFD animals at all time-points compared with both Sham groups. Body mass measurements, both absolute and relative, were lower in SCI-HFD versus SCI-Con groups at 4, 5, and 8 weeks post-surgery and tended to be lower at all time-points. (Please refer to Supplementary Table S1 for complete simple effects testing breakdown between groups).

### Blood glucose

**FIG. 2.**
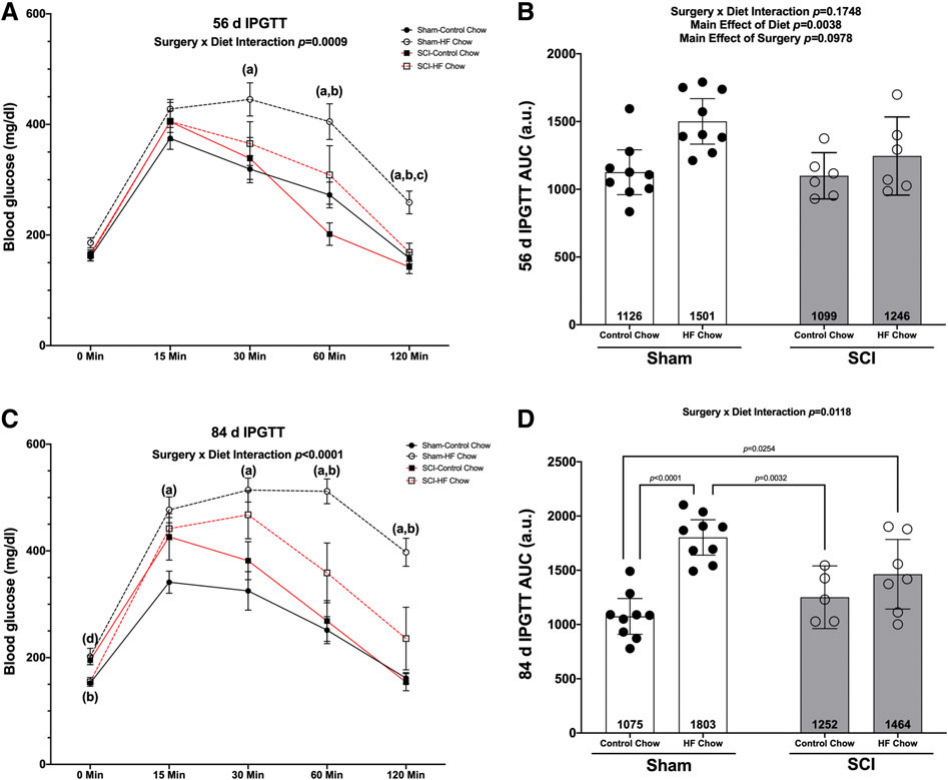
Outcomes of an IPGTT in mice given a Sham or complete transection SCI. **(A)** Mean group responses at 56 days with **(B)** associated AUC values. **(C)** Mean group IPGTT responses at 84 days post-surgery with **(D)** associated AUC values. IPGTT time course values are presented as mean values ± SEM for clarity, whereas AUC are shown as mean values ± a 95% confidence interval. Groups differences as determined by Tukey's multiple comparisons post hoc at each time-point (*p* < 0.05) are denoted as (a) differences compared with Sham-Con, (b) differences compared with SCI-Con, and (c) differences compared with SCI-HFD. Group sizes were: Sham-Con, *n* = 9; Sham-HFD, *n* = 9; SCI-Con, *n* = 4; and SCI-HFD, *n* = 6. AUC, area under the receiver operating characteristic curve; Con, control; HFD, high-fat diet; IPGTT, intraperitoneal glucose tolerance test; SCI, spinal cord injury; SEM, standard error of the mean.

At 56 days post-surgery, there was a Surgery × Diet interaction effect for absolute blood glucose levels (Fig. 2A; *p* = 0.0009) with multiple comparisons testing of pair-wise differences showing elevated levels of blood glucose in the Sham-HFD compared with Sham-Con at 30 (*p* = 0.0241), 60 ( *p* = 0.0218), and 120 min post-injection (*p* = 0.0046). Blood glucose levels in the Sham-HFD group were also elevated compared with SCI-Con at 60 min (*p* = 0.0008) and both SCI-Con (*p* = 0.0018) and SCI-HFD (*p* = 0.0211) at 120 min. There was no interaction effect for total AUC for blood glucose after the IPGTT at 56 days (Fig. 2B; *p* = 0.1748) but there were main effects for greater AUC in HFD versus Con animals (*p* = 0.0038).

At the 84-day time-point, there was a Surgery × Diet interaction effect for absolute blood glucose levels after an IPGTT (Fig. 2C; *p* < 0.0001) with the SCI-Con animals having elevated blood glucose levels at baseline (0 min) compared with Sham-Con (*p* = 0.0145) and SCI-HFD (*p* = 0.0276). Sham-HFD had elevated blood glucose levels compared with Sham-Con and SCI-Con groups at all time-points (baseline: *p =* 0.0502, 15 min: *p =* 0.0032, 30 min: *p =* 0.0031, 60 min: *p <* 0.0001, and 120 min: *p <* 0.0001) and compared with SCI-Con at 60 min (*p =* 0.0040) and 120 min (*p <* 0.0001). There was a Surgery × Diet interaction effect for total AUC for the 84-day IPGTT (Fig. 2D; *p =* 0.0118). Follow-up multiple comparisons testing showed the Sham-HFD animals had greater AUC compared with Sham-Con (*p <* 0.0001) and SCI-Con (*p* = 0.0032), whereas the AUC for SCI-HFD was elevated compared with Sham-Con animals (*p* = 0.0254). There was a strong trend for greater AUC in Sham-HFD compared with Sham-Con (*p* = 0.0603).

### Muscle mass

**FIG. 3.**
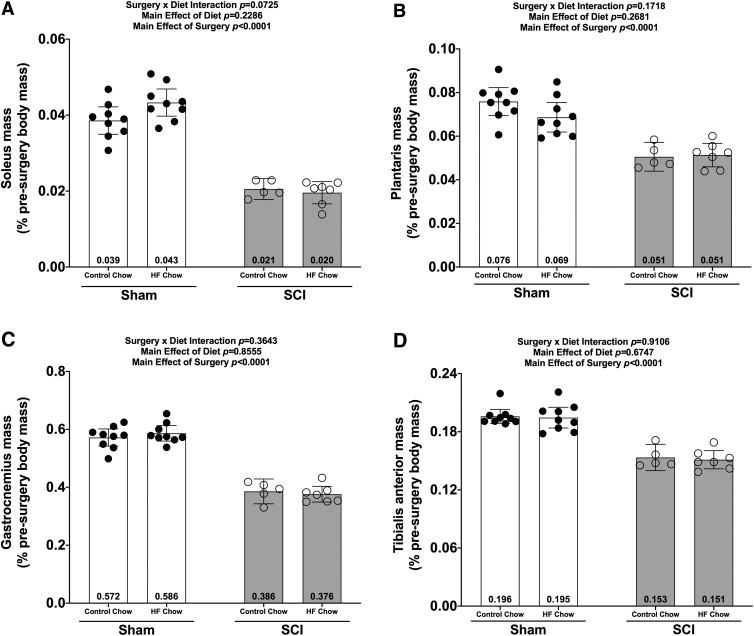
Wet weights of hindlimb **(A)** soleus, **(B)** plantaris, **(C)** gastrocnemius, and **(D)** tibialis anterior 84 days post-surgery were normalized based on pre-operative body weights and plotted as mean values ± a 95% confidence interval. Group sizes were: Sham-Con, *n* = 9; Sham-HFD, *n* = 9; SCI-Con, *n* = 5; and SCI-HFD, *n* = 7. Con, control; HFD, high-fat diet; SCI, spinal cord injury.

There were no Surgery × Diet interaction effects for any of the muscle groups at the 84-day end-point. There were main effects for SCI-induced losses in mass for the soleus (Fig. 3A; *p* < 0.0001), plantaris (Fig. 3B; *p* < 0.0001), gastrocnemius (Fig. 3C; *p* < 0.0001), and TA (Fig. 3D; *p* < 0.0001).

### Liver mass

**FIG. 4.**
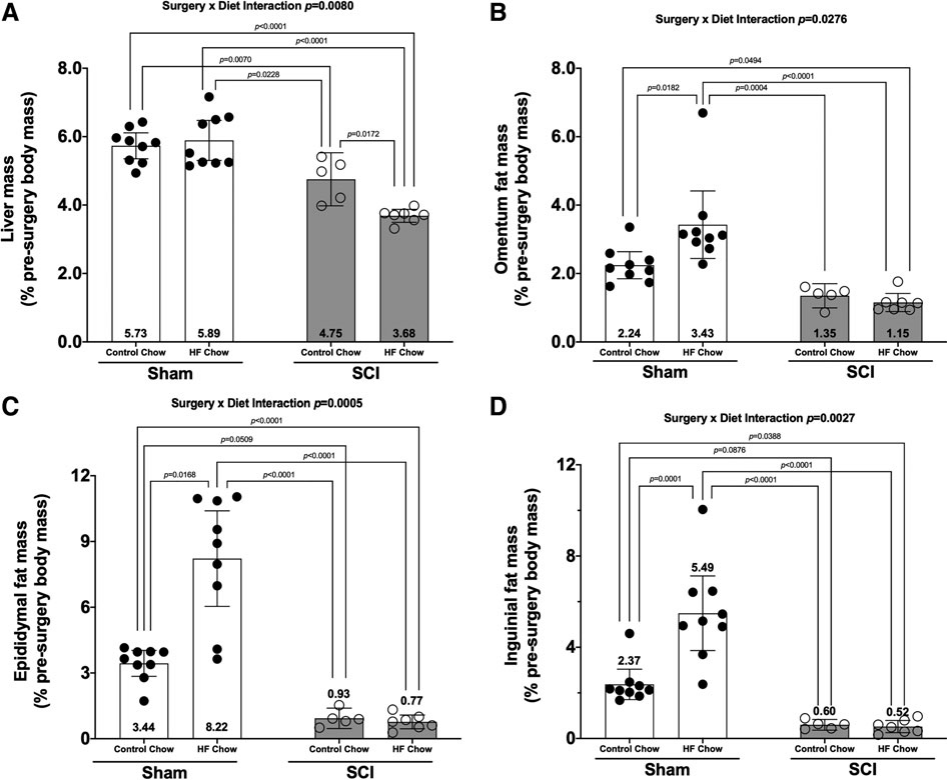
Changes in **(A)** liver weight and **(B)** omentum, **(C)** epididymal, and **(D)** inguinal fat deposit weights 84 days post-surgery. Weights were normalized based on pre-operative body weights and were plotted as mean values ± a 95% confidence interval. Group sizes were: Sham-Con, *n* = 9; Sham-HFD, *n* = 9; SCI-Con, *n* = 5; and SCI-HFD, *n* = 7. Con, control; HFD, high-fat diet; SCI, spinal cord injury.

There was a Surgery × Diet interaction effect for liver mass (Fig. 4A; *p* < 0.0080). Post hoc tests showed reduced liver mass in the SCI-HFD group compared with the Sham-Con (*p* < 0.0001), Sham-HFD (*p* < 0.0001), and SCI-Con (*p* = 0.0172) groups. Additionally, liver mass was lower in the SCI-Con group compared with the Sham-Con (*p* = 0.0070) and Sham-HFD (*p* = 0.0228) groups.

### Fat mass

There was a Surgery × Diet interaction for oFat (Fig. 4B; *p* = 0.0276), eFat (Fig. 4C; *p* = 0.0005), and iFat (Fig. 4D; *p* = 0.0027). Pair-wise comparisons demonstrated the following: Sham-HFD had greater oFat, eFat, and iFat masses versus all other groups (*p =* 0.0182 to <0.0001). When compared with Sham-Con, both SCI groups had lower oFat (vs. SCI-Con: no difference; vs. SCI-HFD: *p* = 0.0494), eFat (vs. SCI-Con: trend, *p* < 0.0509; vs. SCI-HFD: *p* < 0.0001), and iFat (vs. SCI-Con: trend, *p* = 0.0876; vs. SCI-HFD: *p* = 0.0388) masses. No differences in fat mass were present between SCI-Con and SCI-HFD groups.

### Bone mass and morphology

**FIG. 5.**
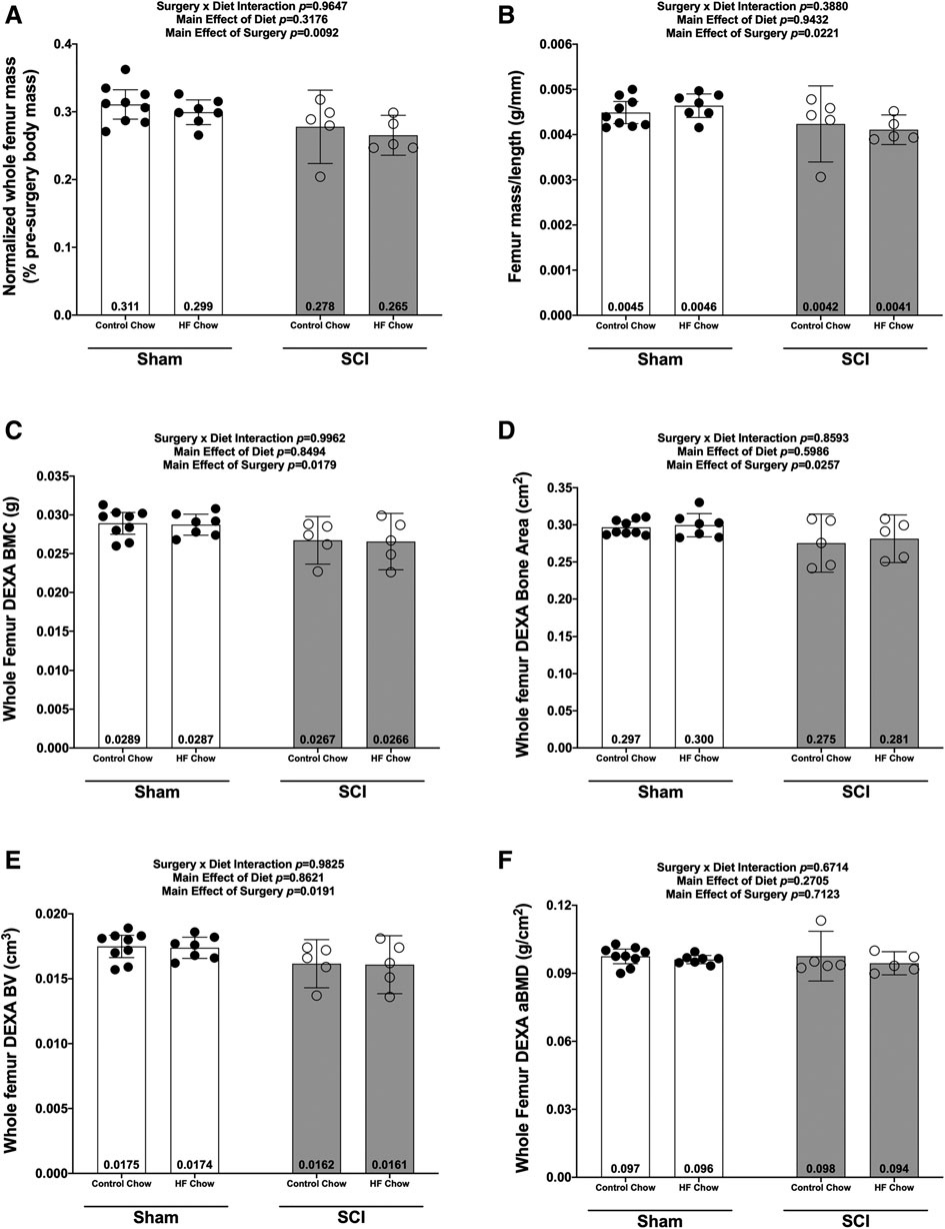
Whole femur mass and DEXA outcomes at 84 days post-sham or complete spinal cord transection with or without a high fat diet. **(A)** Normalized femur mass, **(B)** femur mass/length ratio, **(C)** bone mineral content (BMC), **(D)** bone area, **(E)** bone volume, and **(F)** bone mineral density (BMD). Femur mass was normalized based on pre-operative body weights and all values are plotted as means ± a 95% confidence interval. Group sizes were: Sham-Con, *n* = 9; Sham-HFD, *n* = 8; SCI-Con, *n* = 5; and SCI-HFD, *n* = 5. Con, control; DEXA, dual-energy x-ray absorptiometry; HFD, high-fat diet; SCI, spinal cord injury.

**FIG. 6.**
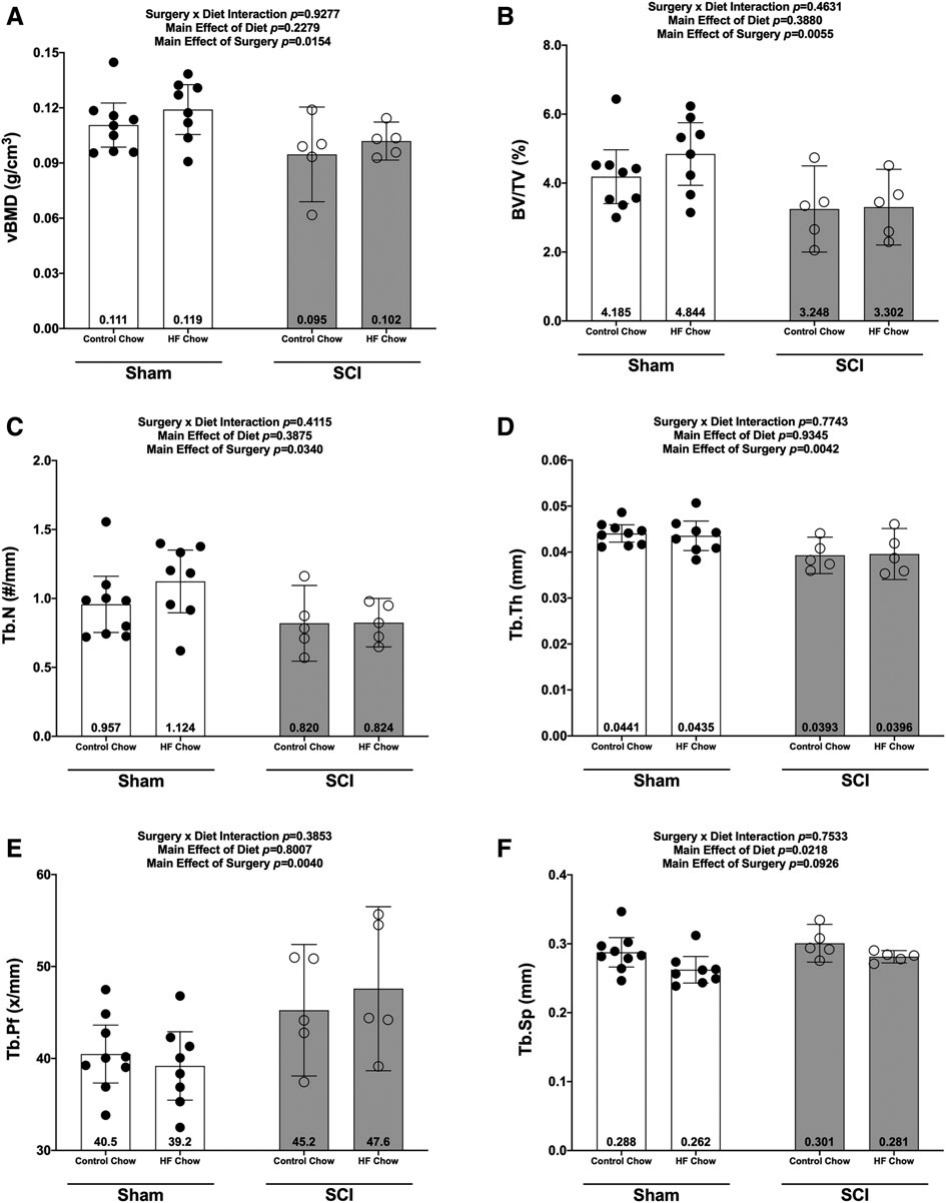
Changes in micro-CT outcomes 84 days after SCI for the distal femoral epiphysis. **(A)** Volumetric bone density (vBMD), **(B)** bone volume/total volume (BV/TV), **(C)** trabecular number (Tb.N), **(D)** trabecular thickness (Tb.Th), **(E)** trabecular pattern factor (Tb.Pf), and trabecular separation (Tb.Sp). All values are plotted as mean ± a 95% confidence interval. Group sizes were: Sham-Con, *n* = 9; Sham-HFD, *n* = 8; SCI-Con, *n* = 5; and SCI-HFD, *n* = 5. Con, control; CT, computed tomography; HFD, high-fat diet; SCI, spinal cord injury.

**FIG. 7.**
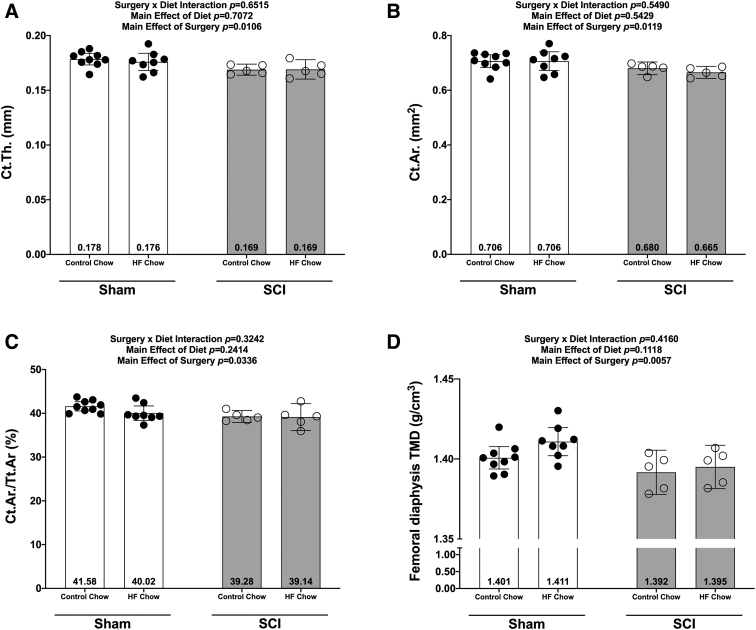
Femoral cortical bone changes at the mid-shaft of the femur at 84 days following a complete spinal cord transection. **(A)** Cortical thickness (Ct.Th), **(B)** cortical area (Ct.Ar), **(C)** cortical area/cortical volume (Ct.Ar/Ct.V), and **(D)** cortical volumetric tissue mineral density (TMD). All values are plotted as mean ± a 95% confidence interval. Group sizes were: Sham-Con, *n* = 9; Sham-HFD, *n* = 8; SCI-Con, *n* = 5; SCI-HFD, *n* = 5. Con, control; HFD, high-fat diet; SCI, spinal cord injury.

There were no Surgery × Diet interactions for bone mass, bone length, or any DEXA- or microCT-derived bone outcomes. However, there were main effects indicating that SCI displayed lower normalized whole femur bone mass (Fig. 5A; *p* = 0.0092) and lower mass:length ratio (Fig. 5B; *p* = 0.0221). DEXA revealed that SCI displayed lower whole femur BMC (Fig. 5C; *p* = 0.0179, bone area (Fig 5D; *p* = 0.0257), and estimated volume (Fig. 5E; *p* = 0.0257), although no difference in whole femur aBMD was detected (Fig. 5F; *p* = 0.7123). High-resolution microCT revealed that SCI groups displayed less cancellous bone at the distal femoral ROI, indicated by lower vBMD (Fig. 6A; *p* = 0.0154), lower BV/TV (Fig. 6B; *p* = 0.0055), lower Tb.N (Fig. 6C; *p* = 0.0340), and lower Tb.Th (Fig.6D; *p* = 0.0042) versus Sham groups. In addition, trabecular pattern factor (Tb.Pf) was higher in SCI animals (Fig. 6E; *p* = 0.0040), indicating that the residual cancellous bone network was less connected than that of Sham animals. At the femoral diaphysis, SCI displayed less cortical bone, indicated by lower Ct.Th (Fig. 7A; *p* = 0.0106), lower Ct.Ar (Fig. 7B; *p* = 0.0119), and Ct.Ar/Tt.Ar (Fig. 7C; *p* = 0.0336) versus Sham groups. Further, femoral diaphysis TMD was lower in SCI versus Sham (Fig. 7D; *p* = 0.0057) groups, indicating the residual cortical bone was less mineralized after SCI.

## Discussion

The findings support several major conclusions: (1) SCI animals display signs of glucose intolerance at 84 days, but not 56 days, after initiating HFD despite a decrease in body mass and lower iFAT, eFAT, and oFAT masses; (2) glucose intolerance was not observed in SCI-Con mice at 56 or 84 days after spinal cord transection, although glucose levels were elevated at baseline and 15 min post-IPGTT in SCI-Con versus Sham-Con at 84 days; (3) SCI reduced the mass of hindlimb muscle, eFAT, oFAT, and iFAT and HFD did not alter this effect; (4) SCI reduced liver mass and feeding HFD to SCI mice further increased liver weight loss; (5) SCI produced cancellous and cortical bone loss and HFD did not alter this effect; and (6) the Sham-HFD group demonstrated glucose intolerance at 56 and 84 days as well as increased eFAT, oFAT, and iFAT mass without a change in liver mass consistent with prior reports.^17,29^

### Emergence of glucose intolerance in HFD-fed SCI mice

A goal of the present study was to test if feeding mice with spinal cord transection a diet known to induce glucose intolerance in neurologically intact mice would result in elevated glucose levels during an IPGTT. One conclusion supported by the data is that glucose intolerance was observed in the SCI-HFD group at 84 days after SCI as indicated by the increased AUC for IPGTT. This outcome is consistent with prior studies of the effects of diets containing 60% fats in otherwise normal male C57BL6 mice.^17,29^ These observations establish HFD feeding as a model for intolerance to carbohydrates in subacute or chronic SCI in mice and may also provide an avenue for obtaining deeper insights regarding the cellular, biochemical, and molecular underpinnings of the pathogenesis of T2DM in persons with SCI.

When considering the glucose intolerance observed in SCI-HFD mice, it is important to note that SCI markedly reduced the mass of all fat deposits examined and that, in contrast to the Sham-HFD group, HFD did not increase the adipose tissue mass after SCI. Thus, glucose intolerance developed in SCI-HFD mice despite markedly reduced adipose tissue deposits. These findings are in marked contrast to body composition changes in patients with SCI who typically gain fat mass,^30,31^ suggesting that caution be used when translating findings from the SCI-HFD mouse model reported here to the clinical SCI population.

The outcomes reported in this study are, in many ways, consistent other studies that have investigated effects of SCI on body composition and metabolic parameters. Mice fed a high-fat high-sucrose diet for 12 weeks prior to a lateral compression SCI at T8-9 then continued on the diet for 30 days after SCI demonstrated higher body weights and reduced glucose tolerance, associated with reduced measures of locomotor function.^32^ However, it is not clear if differences in the aforementioned parameters were present at time of surgery as a result of pre-feeding a high-fat high-sucrose diet for several months before surgery. Rats with a spinal cord transection at T4 demonstrated lower fat mass and reduced glucose and insulin levels at 4 months post-SCI compared with sham controls.^33^ A more recent analysis of spinal cord transected rats demonstrated increased visceral fat mass, a risk factor for perturbances of insulin action,^8,11^ and higher serum triglycerides in animals with a T4 but not a T10 transection.^15^

### Loss of fatty tissue after SCI

The reduced weight of adipose tissue deposits observed after SCI in our study is consistent with that of a report on effects of a T3 spinal cord transection in rats in which it was reported that both brown and white adipose tissue deposits were markedly diminished in size.^33^ Surprisingly, in the study reported herein, SCI-HFD mice did not gain adipose tissue at the sites analyzed. The explanation for this unexpected result is unclear, but it should be noted that although body weights in both SCI-Con and SCI-HFD groups decreased over the first week after surgery, they increased each week thereafter in the SCI-Con group, consistent with our prior work using this mouse SCI model.^22^ Body weights appeared to increase in the SCI-HFD group as well, although less quickly. The gradual rise in body weights over time can best be explained by a net surplus of calories ingested relative to those expended. That body weight did not continue to decrease over time in both SCI-Con and SCI-HFD groups beyond week 1 after SCI argues against inadequate nutritional intake as the basis for the low masses observed for iFAT, oFAT, and eFAT. One alternative mechanism for the decreased mass of fatty tissue deposits after SCI may involve infiltration by inflammatory cells,^11^ which has been proposed as a mechanism driving adipocyte hypoxia and death and which may be an additional factor in pathogenesis of insulin resistance, although this phenomenon is more often observed in markedly enlarged fatty tissue deposits.^11^

The effects of SCI on caloric intake, caloric expenditure, and adipose tissue have been reported for rats. There are discrepancies in findings of these studies suggesting that further research is needed to fully understand how SCI modifies these body composition and physiological parameters in rodents. For example, studies in T4-transected rats have shown slight although statistically significant increased caloric intake and caloric expenditure.^33^ In contrast, in a T10 spinal cord contusion model, cumulative caloric intake of normal chow was reduced in SCI animals compared with Sham controls.

Additionally, a metabolic cage study showed that spinal cord contused rats had reduced caloric intake, and reduced 24-h cage activity levels but elevated caloric expenditure.^34^ Percent body fat was not altered by spinal cord contusion in this study.^34^ Thus, it may be that thoracic contusion injuries create a mild state of hypermetabolism or thermal uncoupling in the chronic stage of SCI in rats. In these spinal cord contused rats, percentage body fat was increased by feeding the SCI rats a diet containing 40% fat, 45% carbohydrate, and 15% protein in association with a non-significant trend toward higher body weights over time when compared with SCI rats feed macronutrient controlled chow with a composition similar to normal rat chow.^34^ It is important to note that, consistent with the current study in mice, all reports in rats show gradual increases in body weight at later time-points indicating a small but significant net excess of caloric intake relative to caloric expenditure.^33,34^

### Effects of SCI on liver weights

An additional unexpected finding was the significantly reduced weights of the liver in SCI-Con mice and further significant reduction of liver weight for SCI-HFD mice. The reasons for these alterations are unclear. Liver weights decline in starved mice, providing one possible explanation for the observed changes in our SCI-Con and SCI-HFD mice.^35^ However, as noted above, the gradual rise in body weight over time in the present study and a prior report in spinal cord transected mice,^22^ as well as in post-SCI rats^33,34^ provide indirect evidence that calorie intake exceeded caloric expenditure, suggesting that alternative explanations should be considered.

One alternative is inflammatory changes in the liver. Sauerbeck and colleagues evaluated effects of a 200-kdyne contusion on liver outcomes in rats and observed evidence of increased liver Kupfer cells and expression of pro-inflammatory cytokines, increased serum levels of a marker of hepatocellular injury, and accumulation of lipids in the liver.^23^ Prior studies of changes in the liver in humans with SCI have used magnetic resonance imaging or abdominal ultrasound^36–38^ have documented that a significant proportion of the SCI population demonstrate imaging evidence of changes compatible with non-alcoholic fatty liver disease (NAFLD) or non-alcoholic steatohepatitis (NASH); these studies have not commented specifically on how liver size compares with that of the able-bodied population. Further investigation is needed to fully elucidate the mechanisms responsible for and biological significance of the low liver weights in our T10 SCI model.

### Effects of HFD on muscle atrophy and sublesional osteoporosis

Several effects of dietary intake of fats on skeletal muscle have been reported. Studies with mice have found evidence of effects of HFDs on skeletal muscle that include reduced strength^39^ and alterations in mitochondrial function.^40^ In the present study, muscle mass was not altered by feeding SCI mice a HFD. This result is consistent with observations in spinal cord contused rats that a diet containing 40% fat did not alter lean tissue mass.^34^

Bone is recognized as an insulin-responsive tissue and additional site for glucose uptake due to the continual activity of osteoclasts and osteoblasts in regulating bone turnover throughout the lifespan.^41^ Osteoblasts secrete osteocalcin into the extracellular matrix, which is decarboxylated by the low pH occurring during osteoclastic bone resorption and released into the circulation. Subsequently, under-carboxylated osteocalcin can signal pancreatic β cells to produce insulin via cell-surface Gprc6a receptors, an effect that has significant implications for systemic glucose homeostasis.^42^ In our model, we did not observe any diet-induced bone phenotype in either SCI-HFD or Sham-HFD mice, despite others reporting that HFD produced a low trabecular bone mass phenotype in various rodent models.^24^ It is unclear why no bone loss was observed in the Sham-HFD group. It should also be noted that any potential HFD-induced bone loss in the SCI-HFD group may have been overshadowed by the dramatic bone deficits occurring as a result of SCI. In this regard, the effects of disuse and/or SCI on skeletal insulin-responsiveness, glucose disposal, and release of under-carboxylated osteocalcin remain relatively unknown and are an exciting area for future investigation.

### Study limitations

Our study has several limitations. Because patients with SCI typically gain fat mass with time after injury,^30,31^ the decrease in fatty tissues in SCI mice limits clinical translation of the findings. In addition, a spinal cord transection was used that, although advantageous for its uniform, complete, and reproducible paralysis does not fully recapitulate the more common complex clinical lesion. It is quite possible that a lesion from a crush or contusion SCI has somewhat different outcomes and systemic effects due to sparing of white matter tracts across the area of injury. We did not make formal recordings of food intake, so it is possible that some of the outcomes we note are due to altered caloric intake. Lastly, we cannot exclude the possibility that older mice (e.g., 6 and 12 months old) would respond differently from younger mice to HFD under sham and SCI conditions.

## Conclusion

In C57B6 mice, a mid-thoracic spinal cord transection reduces weights of eFat, oFat, and iFat adipose pads, liver, and muscle and elevates fasting blood glucose. Feeding spinal cord transected mice a diet containing 60% long-chain fatty acids induces glucose intolerance within 84 days of surgery. The marked bone loss and muscle atrophy occurring after spinal cord transection is not altered by a HFD, although the data do not permit any conclusions about whether the rate of bone loss might have differed. This model may be useful in elucidating cellular, physiological, and molecular changes by which metabolism of fats and carbohydrates is dysregulated after SCI and to evaluate the potential efficacy of therapies to improve metabolic outcomes after SCI.

## Supplementary Material

Supplemental data

Supplemental data

## Abbreviations Used

aBMD: areal bone mineral density
ANOVA: analysis of variance
AUC: area under the receiver operating characteristic curve
BMC: bone mineral content
BV/TV: cancellous bone volume
Con: control
Ct.Ar: cortical bone area
Ct.Ar/Tt.Ar: cortical area fraction
Ct.Th: cortical thickness
DEXA: dual-energy x-ray absorptiometry
eFat: epididymal
HFD: high-fat diet
inguinal: iFat
IPGTT: intraperitoneal glucose tolerance test
microCT: microcomputed tomography
NAFLD: non-alcoholic fatty liver disease
NASH: non-alcoholic steatohepatitis
oFat: omentum
ROI: region of interest
SCI: spinal cord injury
SEM: standard error of the mean
T2DM: type 2 diabetes mellitus
TA: tibialis anterior
T.Ar: total area inside the periosteal envelope
Tb.N: trabecular number
Tb.Pf: trabecular pattern factor
Tb.Sp: trabecular separation
Tb.Th: trabecular thickness
vBMD: cancellous volumetric bone mineral density
vTMD: cortical volumetric tissue mineral density
