# Supplementary material for: Effects of a High-Fat Diet on Tissue Mass, Bone, and Glucose Tolerance after Chronic Complete Spinal Cord Transection in Male Mice
Source: Neurotrauma Rep. Author manuscript; Available in PMC 2021 Jul 1. (PMC8240892; doi:10.1089/neur.2020.0014)
Supplement: Supp_Fig_1 [file NIHMS1695160-supplement-Supp_Fig_1.pdf]

Supplementary Data

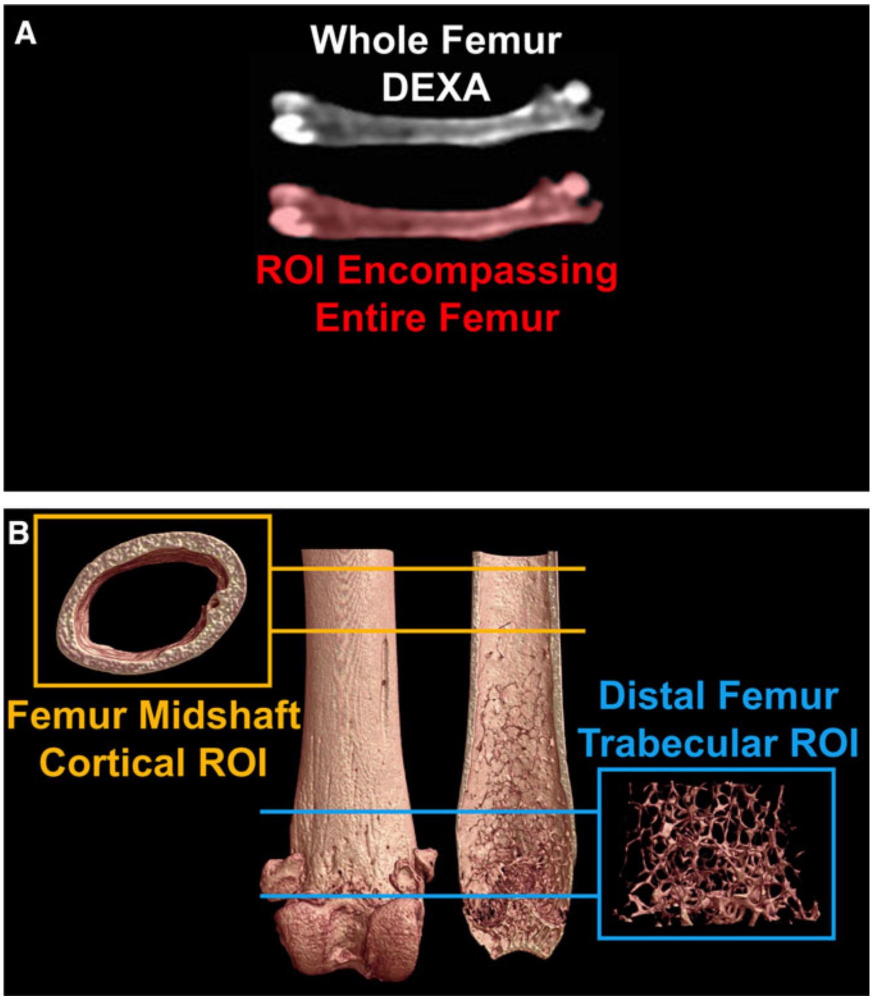

SUPPLEMENTARY FIG. S1. **(A)** Representative dual-energy x-ray absorptiometry (DEXA) and microcomputed tomography (microCT) derived images of the whole femur and selected regions of interest (ROIs). The DEXA ROI (outlined in red) encompassed the entire femur. **(B)** The microCT trabecular ROI (outlined in blue) began 0.5 mm proximal to the distal femoral metaphysis growth plate and encompassed 1.5 mm in the proximal direction. The microCT cortical ROI (outlined in orange) encompassed 1.0 mm surrounding the femoral diaphysis.
